# A Novel Mouse Model of Penetrating Brain Injury

**DOI:** 10.3389/fneur.2014.00209

**Published:** 2014-10-22

**Authors:** Ibolja Cernak, Ian D. Wing, Johan Davidsson, Stefan Plantman

**Affiliations:** ^1^Military and Veterans’ Clinical Rehabilitation Research, University of Alberta, Edmonton, AB, Canada; ^2^Johns Hopkins University Applied Physics Laboratory, Laurel, MD, USA; ^3^Division of Vehicle Safety, Chalmers University of Technology, Göteborg, Sweden; ^4^Department of Neuroscience, Karolinska Institutet, Stockholm, Sweden

**Keywords:** traumatic brain injury, models of injury, penetrating ballistic-like brain injury, animal studies, gliosis, neurodegeneration, histology

## Abstract

Penetrating traumatic brain injury (pTBI) has been difficult to model in small laboratory animals, such as rats or mice. Previously, we have established a non-fatal, rat model for pTBI using a modified air-rifle that accelerates a pellet, which hits a small probe that then penetrates the experimental animal’s brain. Knockout and transgenic strains of mice offer attractive tools to study biological reactions induced by TBI. Hence, in the present study, we adapted and modified our model to be used with mice. The technical characterization of the impact device included depth and speed of impact, as well as dimensions of the temporary cavity formed in a brain surrogate material after impact. Biologically, we have focused on three distinct levels of severity (mild, moderate, and severe), and characterized the acute phase response to injury in terms of tissue destruction, neural degeneration, and gliosis. Functional outcome was assessed by measuring bodyweight and motor performance on rotarod. The results showed that this model is capable of reproducing major morphological and neurological changes of pTBI; as such, we recommend its utilization in research studies aiming to unravel the biological events underlying injury and regeneration after pTBI.

## Introduction

Penetrating traumatic brain injury (pTBI) occurs when an object impacts the head with sufficient energy to penetrate the skin, skull, and meninges and inflict injury directly to the brain tissue. Historical case studies of pTBI provided the best known information about human neurological functions. For example, the descriptions of injuries suffered by Phineas Gage (1), the soldier known in the medical literature under the pseudonym Zasetsky (2), and the patient known as N.A. (3) have greatly contributed to our understanding of the neurological mechanisms underlying normal or pathological sensation or cognitive processing. In general, pTBI is a severe type of injury, particularly prevalent in warzones (4, 5) and in areas with high incidence of gun-related violence (6). In contrast to closed head injury, pTBI involves direct laceration of brain tissue, often complicated by hemorrhage, edema, inflammation, higher risk of coagulopathy (7), and post-traumatic seizures (8). The presence of foreign objects such as bone- or missile-fragments might promote post-traumatic infection and worsen the outcome after pTBI (9, 10). Due to the complexity of severity of pTBI, special guidelines have been developed for its management (11).

To date, the number of animal models reproducing penetrating head injury is very limited. Cat (12), dog (13), monkey (14), and sheep (15) have all been used for this purpose, but none of these models are currently in routine use. Although anatomical and physiological properties of larger animal models might be more comparable to humans, rodents have the advantage of being easier to handle and due to their relatively small cost permit repetitive measurements of morphological, biochemical, and cellular parameters (16). Moreover, standardized and reproducible ways to measure outcome in terms of behavior are mainly available for rodents (17).

At current, there are only two rodent pTBI models: (1) the penetrating ballistic-like brain injury (PBBI) model in rats developed by Williams et al. (18), which simulates the large temporary cavity caused by energy dissipation from a penetrating bullet-round using an inflatable penetrating probe (18); and (2) our recently developed pTBI model (19) using a pellet accelerated by a modified air-rifle and hitting a probe, which then penetrates the brain of an anesthetized rat. The induced injury causes neuronal and axonal degeneration, blood–brain barrier (BBB)-defects, reactive gliosis, and functional impairment (19), and significantly differs from blast- and rotation-induced injuries in terms of induction of gene expression in the injured brain (20). Both above-mentioned rodent pTBI models have been designed for rats; however, bearing in mind some obvious advantages of using mice as experimental animals, such as gene-manipulation and smaller housing requirements (21), we redesigned and adapted our rat model for use with mice. This paper describes the initial technical testing and biological characterization of this new model.

## Materials and Methods

### Design and adaptation of the pTBI device

**Figure 1:**
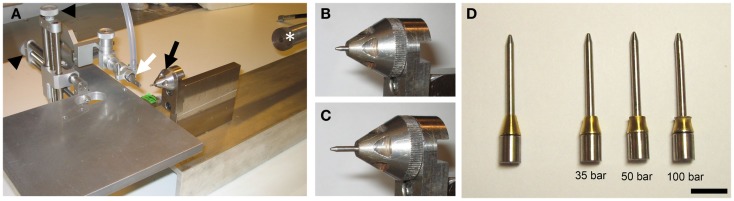
**The penetration device**. **(A)** Photo of the penetration rig showing the mouthpiece (white arrow), probe holder (black arrow), stereotactic manipulators (black arrowheads), and air-rifle barrel (white asterisk). **(B,C)** Close-up of the probe holder and probe before **(B)** and after **(C)** impact of pellet. Note the probe displacement (penetration) after impact **(C)**. **(D)** Photo of probes after impact at loading-pressures of 35-, 50-, and 100-bar. Note deformation of the brass ferrule (scale bar = 10 mm).

The rat version of the pTBI rig has been described previously (19). Briefly, a lead pellet (Accupell, Crossman, Bloomfield, NY, USA) is accelerated from a modified air-rifle (CNC-Process AB, Hova, Sweden) (Figure 1A) connected to a tank filled with compressed air. This pellet then hits a probe sitting in a holder (Figures 1B,C), and a brass ferrule (Swagelok, Solon, OH, USA) fitted around the probe controls the depth of penetration. The injury severity is varied by adjusting the air-rifle pressure. Given the smaller size of the mouse brain, which is approximately 6 mm from the top of the cortex to the median eminence in the coronal plane at bregma −2.5 mm (22) as compared to approximately 10 mm for rats (23), we manufactured probes [diameter: 2 mm and weight 1.48 g (Figure 1D)] that reach a penetration depth of 3.55 mm before the ferrule, acting as a stopper, is engaged. Experience from the rat model showed that a probe with a pointed tip causes less tissue destruction than a probe with a blunt tip. Consequently, our mouse probes were made with a pointed tip. The previous experimental setup for rats with these two alterations induced injuries in mice similar to those seen in rats at 50-bar (5 MPa) loading-pressure (data not shown). In this study, we used the same injury severity criteria, determined by histology, as in our previous characterization (19). We chose the injury severity induced by 50-bar loading-pressure as the moderate injury level. A loading-pressure of 35-bar was considered a mild injury and the 100-bar loading-pressure was chosen as the upper limit inducing severe pTBI.

### Penetration depth and probe velocity

The depth of penetration was measured with a slide caliper, before and after the pellet impact. To capture the probe velocity at the impact (i.e., when the probe was impacted by the pellet but did not penetrate the brain), we used a Phantom V10 video camera (Vision Research, Wayne, NJ, USA) at 30,000 frames per second. Video images were analyzed frame-by-frame, and the velocity of the probe was determined.

### Gel indentation measurements

Ballistics gel (Gelatin innovations, Schiller Park, IL, USA) was prepared according to the manufacturers’ instructions.^1^ Different concentrations have been tested in our lab to achieve the gel’s best biological fidelity with the brain tissue, which have been reproduced by a 7% gel. The gel was cast in a transparent box, and video recordings were made using similar settings as for probe velocity measurements described in the previous section. Still images of maximum cavitation were used to trace the cavity profile, and the volume was determined using Cavalieri’s volume estimation: *V* = Σ *t*(π*r*^2^), where *V* is the total volume of the cavity, achieved by adding the individual volumes of the disks [thickness (*t*) used were 0.5 mm and radius (*r*) from video image analysis]. Videos of gel indentation can be found in the Supplementary Material.

### Animals and injury induction

All protocols involving the use of animals were approved by the Johns Hopkins University Animal Use Committee. Male, young adult C57/Bl6 mice weighing between 25 and 28 g were used throughout the study. Mice were kept on a 12 h light/dark cycle, and provided with food and water *ad libitum*. For surgery, mice were initially anesthetized with 4% and then with 1–1.5% isoflurane for maintenance. The anesthetic was evaporated in a gas mixture containing 30% oxygen/70% nitrous oxide and applied through a nose-mask. A midline incision was made through the skin and periosteum, and a burr-hole of 2.7 mm in diameter was drilled with its center 1.5 mm lateral, and 1.5 mm posterior to bregma. The animal was thereafter placed in a stereotactic frame (David Kopf Instruments, Tujunga, CA, USA) and positioned so that the impactor probe was positioned directly above the dura exposed by the burr-hole. Penetration was performed, and the mice were removed from the frame; the scalp was sutured and mice were given a subcutaneous injection of a mixture containing buprenorphine (0.05 mg/kg) and carprofen (5 mg/kg) in 0.5 ml of saline, and thereafter returned to their cage to recover. The number of animals used are summarized in Table 1.

**Table 1:** **Number of animals in the different experimental groups**.

Number of animals	Procedure	Survival	Analysis
15	pTBI (five per severity level)	24 h	Histology
5	Craniotomy	24 h	Histology
15	pTBI (five per severity level)	72 h	Histology
21	pTBI (seven per severity level)	7 days	Weighing rotarod histology
7	Craniotomy	7 days	Weighing rotarod histology

### Tissue harvesting and sectioning

Mice were sacrificed by an overdose of pentobarbital and transcardially perfused with Tyrode’s solution, followed by fixative containing 4% formaldehyde in phosphate buffer (pH 7.0) (APL, Kungens Kurva, Stockholm, Sweden). The brains were removed, post-fixed for 2 h at 4°C, rinsed in 0.01 M PBS and transferred to a 0.01 M PBS solution containing 10% sucrose and stored overnight at 4°C. Subsequently, brains were frozen and then cut in coronal sections (14 μm thickness) starting at bregma −1.5 mm according to Ref. (22), using a cryostat (Microm International Gmbh, Walldorf, Germany). Sections were thaw-mounted onto gelatine-coated slides and stored at −20°C until staining. For all histological analyses a minimum of five sections separated by 70 μm were used.

### Hematoxylin and eosin staining

For general brain pathology, sections were stained in hematoxylin (Histolab, Göteborg, Sweden) for 15 min; rinsed in water (15 min); stained with eosin (Sigma-Aldrich, St. Louis, MO, USA) for 2 min; dehydrated through a series of increasing ethanol concentrations; and finally immersed in xylene for 15 min and cover-slipped with Entellan (Merck, Darmstadt, Germany). For image analysis, pictures were imported into the Image J software (NIH).^2^ The lesioned area was traced manually after calibration against an internal scale bar and measured using the area-function.

### Fluoro-jade staining

To detect degeneration of cortical neurons, sections were stained with Fluoro-Jade C (Millipore, Billerica, MA, USA) according to the manufacturers’ instructions. Briefly, slides were immersed in 80% EtOH with 1% NaOH for 5 min, followed by 2 min in 70% EtOH, 2 min in distilled water, and incubated in 0.06% potassium permanganate solution for 10 min. Slides were subsequently rinsed in water, transferred to a 0.0001% solution of Fluoro-Jade C in 0.1% acetic acid. The slides were then rinsed in distilled water, air dried, and cleared in xylene and cover-slipped with Entellan (Merck).

### Immunohistochemistry

The slides were incubated with primary antibodies overnight at 4°C. The following antibodies were used: anti-Iba1 to label macrophages/microglia (1:400, Wako Pure Chemical Industries, Ltd., Osaka, Japan), anti-GFAP to label astrocytes (1:200, Sigma-Aldrich), and anti-β-amyloid precursor protein (βAPP) as a marker for early axonal damage (1:200, Invitrogen, Camarillo, CA, USA). After three 10-min rinses in 0.01 M PBS, the sections were incubated for 1 h at room temperature with Cy-2-secondary antibodies (1:200, Jackson Immunoresearch, PA, USA) and/or Cy3-secondary antibodies (1:400, Jackson Immunoresearch, PA, USA). For IgG-staining, sections were incubated with anti-rat Cy-2 conjugated antibodies (1:400, Jackson Immunoresearch, PA, USA) overnight, rinsed in PBS, and cover-slipped. Sections were examined using a Nikon E600 microscope (Nikon, Shinjuku, Japan) with appropriate filter settings. Images were captured using a Nikon Digital Sight DS-U1 (5 megapixel) camera, controlled with Nikon EclipseNet software. For image densiometry analysis, pictures were imported into the Image J software, converted to gray-scale and eight-bit format, and quantified using the area fraction function. Slides used for quantification were blind-coded prior to analysis.

### Motor performance

Using the rotarod test, motor scoring was performed before, and at 1, 2, 3, 4, 5, 6, and 7 days after injury. This test is one of the most sensitive tests to detect motor deficits in rodent brain injury (24). Briefly, the animals were placed on a rotarod device (Ugo Basile, Comerio, Varesi, Italy) and permitted to explore the rotarod at constant speed of 5 rpm for 30 s. Then the mice experienced a period during which, the drum was accelerated to 50 rpm over a course of 4 min. Mice were trained for 3 days prior to injury. The rotarod test was performed by measuring the length of time each animal was able to maintain its balance walking on top of the drum. Trials ended when the animal either fell off the rod or clung to the rod as it made one complete rotation. Each mouse was tested three-times and the average was recorded (25). All behavioral analyses were done blindly with regard to injury/control.

### Statistical analyses

Statistical analyses were carried out using GraphPad Prism 5.0 (GraphPad Software, La Jolla, CA, USA). Data were tested for normality using the Column Statistics function and passed the Kolmogorov–Smirnov test. Multiple group comparisons were performed using one-way ANOVA followed by Bonferroni’s test. Repeated measures ANOVA followed by Bonferroni’s test was used for weight and rotarod data.

## Results

### Impact characterization

**Figure 2:**
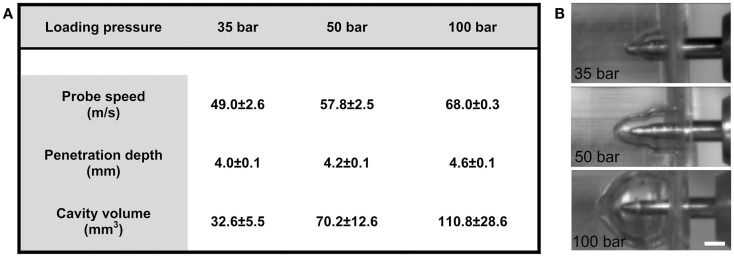
**Technical tests of penetration rig**. **(A)** Table showing the relationship between loading-pressure and maximum speed of probe, depth of penetration, and volume of temporary cavity after indentation in ballistic gel (data presented as mean ± SD, *n* = 3–5 in each condition). **(B)** Images from high-speed videos used for quantification of temporary cavity (scale bar = 2 mm).

The motion of the probe, from initial position to when the ferrule was engaged, was 3.55 mm. Following impact by pellet, the depth of the penetration in ballistics gel was 3.99 ± 0.05 mm with the 35-bar loading-pressure; 4.23 ± 0.03 mm for 50-bar; and 4.61 ± 0.10 mm for 100-bar (Figure 2A). The maximum speed was 49.0 ± 2.6 m/s (35-bar loading-pressure); 57.8 ± 2.5 m/s (50-bar); and 68.0 ± 0.3 for 100-bar loading-pressure (Figure 2A). Maximum cavity volume was 32.6 ± 5.5 mm^3^ (35-bar); 70.2 ± 12.6 mm^3^ (50-bar); and 110.8 ± 28.6 mm^3^ (100-bar) (Figures 2A,B). The weight of the probe was 1.48 g, which would (using the maximum speed for different pressures given in Figure 2A and the formula *e* = 1/2 *mv*^2^) translate into kinetic energy of 1.78 J for 35-bar, 2.50 J for 50-bar, and 3.42 J for 100-bar loading-pressure.

### Survival

We found that the choice of the anesthetic agent (i.e., isoflurane) profoundly influenced the survival of mice. Initially, we planned to use a mixture of fentanyl/fluniasone and midazolam (Hypnorm and Dormicum), as we used in our rat pTBI model (19). However, the small pilot study we conducted to find the optimal experimental setting showed that the survival rate of mice was very low with this regimen of anesthesia, i.e., approximately 50% in all conditions. Hence, we therefore switched to isoflurane anesthesia shown successful in our previous experiments (25–27) and increased the survival rate to 100% in all conditions. This observation is in line with the notion by Statler and co-workers (28), who showed that the use of isoflurane was associated with better cognitive recovery and increased neuronal survival compared to several other common anesthetics after controlled cortical impact (CCI) head injury.

### Histopathology of tissue damage

**Figure 3:**
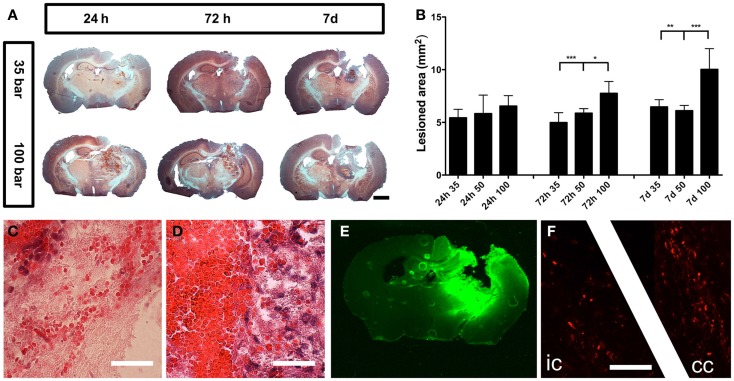
**General pathology**. **(A)** Hematoxylin and eosin staining of mice brains subjected to pTBI with 35 and 100-bar loading-pressure, at different time-points post-injury (scale bar = 2 mm). **(B)** Graphs of the injured area at 24, 72 h, and 7 days post-injury. Data expressed as mean ± SD (*n* = 5 in each group). *p < 0.05, **p < 0.01, ***p < 0.001 (ANOVA followed by Bonferroni’s test. **(C)** Scattered erythrocytes 24 h post-injury. **(D)** Massive accumulation of erythrocytes, surrounded by macrophage-rich tissue 7 days after injury (scale bar = 25 μm). **(E)** IgG-staining showing BBB-defects 24 h after pTBI. **(F)** βAPP-positive axon profiles in the internal capsule “ic” and corpus callosum “cc” 24 h after pTBI (scale bar = 50 μm).

The injury caused severe damage to the lateral and medial parietal cortices, corpus callosum, hippocampus, and several parts of the posterior thalamus (Figure 3A). The injury was progressive, and at 7 days post-injury, the initial stage of cavity formation became visible (Figure 3A). The area of injury caused by the 100-bar loading-pressure was significantly larger than the size of damages caused by the 35- or 50-bar loading-pressures, at both 72 h and 7 days post-injury (Figure 3B). While the erythrocytes in the brain showed a scattered pattern at 24 h post-injury (Figure 3C), at 7 days post-injury, they were found in a confined location, surrounded by dense, macrophage-rich, and tissue (Figure 3D). Positive IgG-staining (Figure 3E) indicating BBB-disruption was found throughout the lesion and largely confined to the ipsilateral side of the brain. Positive βAPP-staining indicating axonal damage was observed in white matter tracts (corpus callosum and the internal capsule) at 24 h post-injury (Figure 3F).

### Neuronal degeneration

**Figure 4:**
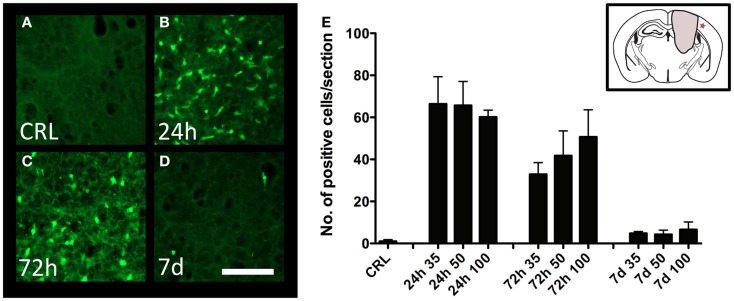
**Neuron degeneration**. Fluoro-Jade staining in the perilesional area in uninjured **(A)** and injured **(B–D)** cortex. All images showing cortex from mice injured at 100-bar severity. Star in insert in **(E)** indicates the area depicted in **(A–D)** (scale bar = 50 μm). **(E)** Graph showing the number of Fluoro-Jade-labeled neuronal profiles (*n* = 5–6 in each group, data expressed as mean ± SD).

Degeneration of cortical neurons was assessed by Fluoro-Jade staining. While no staining was observed in control animals (Figure 4A), there was a significant increase in the number of labeled neurons at 24 h post-injury (Figures 4B,E), with diminishing numbers at 72 h (Figures 4C,E), and 7 days (Figures 4D,E). The trend was similar in all injury severities, and no statistical difference between severity levels could be detected (Figure 4E).

### Reactive gliosis

**Figure 5:**
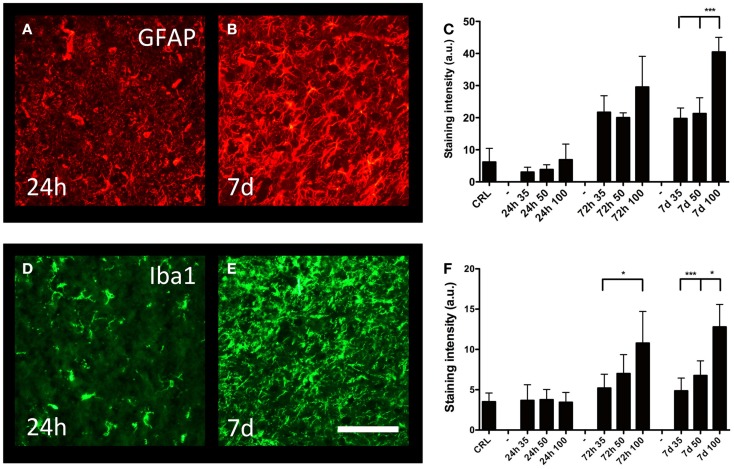
**Astro- and microgliosis**. GFAP-staining in the perilesional area (same area as in Figure 4E), at 24 h **(A)** and 7 days **(B)** post-injury, both with 100-bar loading-pressure. **(C)** Graph showing the staining intensity at 24, 72 h, and 7 days post-injury for 35-, 50-, and 100-bar loading-pressure. Data expressed as mean ± SD (*n* = 5 in each group, ***p < 0.001 ANOVA followed by Bonferroni’s test. Iba1-staining **(D,E)** after 100-bar loading-pressure. **(F)** Graph of staining intensity at 24, 72 h, and 7 days post-injury for 35-, 50-, and 100-bar loading-pressure. Data expressed as mean ± SD (*n* = 5 in each group). *p < 0.05, **p < 0.01, ***p < 0.001 (ANOVA followed by Bonferroni’s test). Scale bar = 50 μm

Cortical gliosis was quantified in the peri-lesion area by image densitometry of GFAP (Figures 5A,B) and Iba1-staining (Figures 5D,E) intensity. An increase in GFAP-staining could be detected after 72 h; after that, the staining intensity further increased and remained so to the end of the observation period (7 days post-trauma) (Figure 5C). At 7 days post-injury, gliosis induced by the 100-bar loading-pressure demonstrated a significantly higher GFAP immunoreactivity compared to 35 or 50-bar loading-pressure-induced changes; nevertheless, this was not the case at 24 or 72 days after the injury. The changes of macrophages/microglia showed a similar time-course of activation (Figure 5F), with the exception that the 100-bar loading-pressure-induced injury severity was significantly higher than those caused by the 35-bar loading-pressure at 72 h post-injury.

### Bodyweight

**Figure 6:**
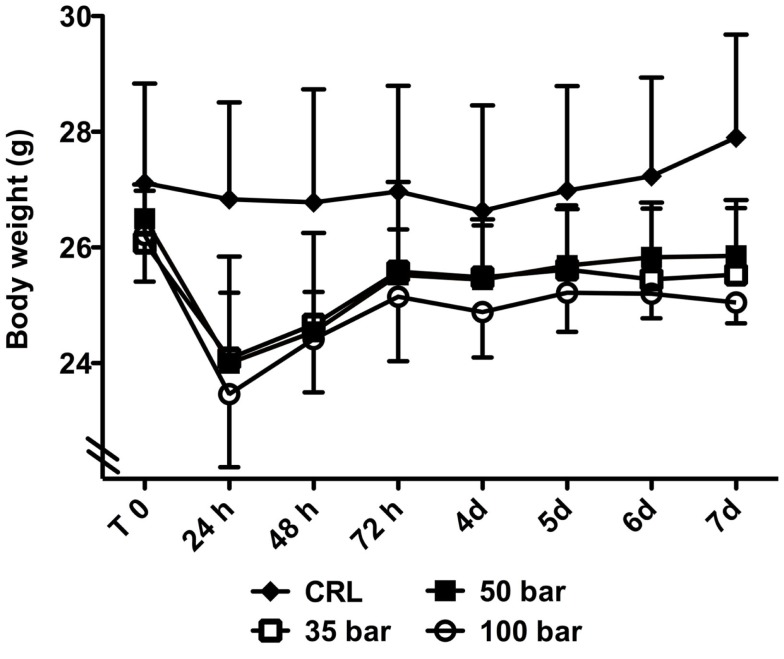
**Weight-loss post TBI**. Graph showing the bodyweight immediately prior to (T0) and at various time-points post TBI. Data expressed as mean ± SD (*n* = 7 in each group). No significant difference between the injured groups could be detected (CRL stands for sham-exposed controls).

At 24 h after injury, sham control animals had lost 1.0 ± 3.1% of their bodyweight, whereas injured animals had lost 7.6 ± 4.4% (35-bar), 9.5 ± 6.4% (50-bar), and 10.4 ± 4.5% (100-bar) (Figure 6). After that, there was a gradual increase in weight in all groups, and at the end of the experiment (7 days post-injury), sham control mice had gained 2.9 ± 3.4% compared to a loss of 2.1 ± 2.3% for the 35-bar group, 2.4 ± 2.2% for the 50-bar group, and 4.3 ± 3.6% for the 100-bar group (*n* = 6 in all groups).

### Motor function

**Figure 7:**
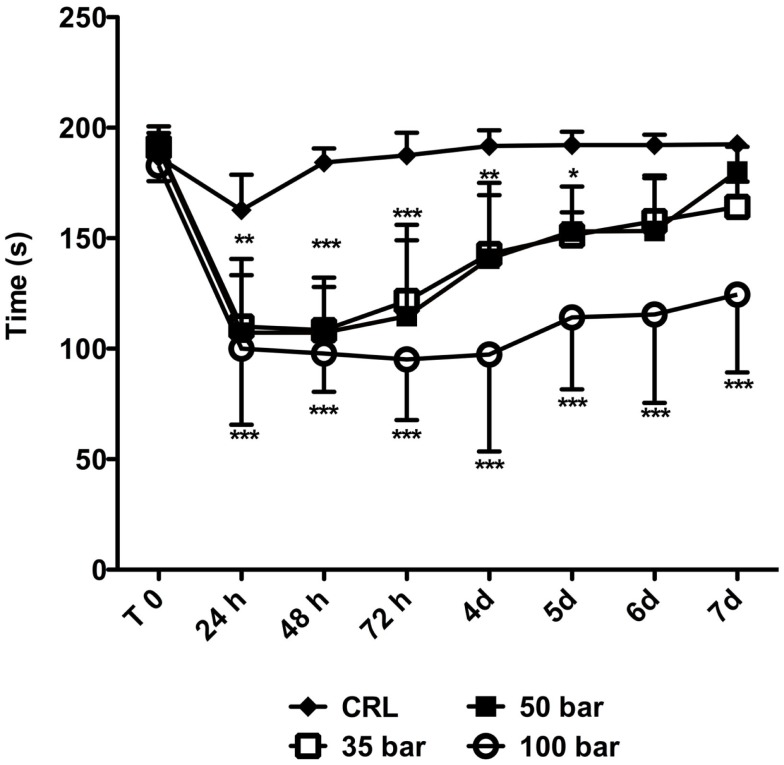
**Rotarod-performance post TBI**. Graph showing the time on rotarod immediately prior to (T0) and at various time-points post TBI. Data expressed as mean ± SD (*n* = 7 in each group). *p < 0.05, **p < 0.01, ***p < 0.001 (repeated measures ANOVA followed by Bonferroni’s test compared to control).

Animals were tested immediately prior to injury (“T0” in Figure 7), and then daily up to 7 days post-injury. The average “T0” time was 187 ± 6 s. At 24 h post-injury, the average time the sham control group (*n* = 6) spent on the rotarod was 163 ± 16 s followed by a rapid improvement to the “T0” level. At 24 h post-injury, the time spent on the rotarod decreased to 110 ± 23 s for the 35-bar group, to 107 ± 33 s for the 50-bar group, and 100 ± 34 s for the 100-bar group (*p* < 0.01 for 35 and 50-bar, *p* < 0.001 for 100-bar compared to control, *n* = 7 in all groups). The 35 and 50-bar groups then gradually regained function, and at day 6 post-injury, they were not statistically different from the control group. However, the 100-bar group still had severe functional deficits by 7 days, 125 ± 30 s (*p* < 0.001).

## Discussion

### Mice as model organism in experimental TBI research

Several TBI models, originally developed for other species, have been adapted for use in mice such as the fluid-percussion (29), CCI (30), weight-drop (25, 31, 32), and the shock tube-generated blast-induced neurotrauma (27, 33) models.

However, for mice, the only routinely used model of pTBI is the stab wound, commonly performed using a scalpel or a needle. Although this model is useful for detailed investigations of cellular reactions such as axotomy, astro- and microgliosis, or induction of inflammatory mediators, it bares little resemblance to penetrating TBI seen in the clinic (21). Despite several obvious differences between the brains of humans and rodents in general that should not be overlooked in experimental TBI research (16, 34–36), gene-altered mice have been used to investigate a number of biochemical pathways and genetic polymorphisms of importance for outcome after TBI (21, 37). We are therefore confident that our new model will contribute to a greater understanding of the injury progression of, and outcome after, penetrating TBI.

### Performance of the pTBI device

Our technical characterization showed that by using three different loading-pressure levels, we were able to produce three different levels of impact severity as determined by speed, penetration depth, and cavity formation (Figures 2A,B). The volume of an adult C57/Bl6 mouse brain is approximately 453 mm^3^ (38), which would indicate that the temporary cavity observed in our gel indentation experiments would take up approximately 7% (35-bar), 15% (50-bar), or 27% (100-bar) of the mouse brain volume. However, in addition to possible difference in material properties, the temporary cavity observed after pTBI is greatly modified by the irregular shape of the cranial vault and the connective tissue surrounding the brain (39), which makes extrapolations from the gel indentation observations to the *in vivo* situation rather uncertain. This experiment did, however, confirm that the energy transferred to the surrounding environment (gel or brain tissue) is related to the force used to cause the injury; thus injury is easily scalable (16).

In experiments using cats, Carey and co-workers (12) found that a missile fired at the brain with a kinetic energy of 2.50 J was fatal in two–thirds of all cases, and with 1.40 J, fatality was around 40%. However, in our model, all injury levels (1.78 J for 35-bar, 2.50 J for 50-bar, and 3.42 J for 100-bar) were associated with a complete level of acute phase survival. One of possible explanations is that the kinetic energy in our model was transferred upon impact not only to the brain tissue, but also to the brass ferrule, as evident by the deformation of the ferrule (Figure 1D). The exact proportions of energy transferred to ferrule and brain tissue remains to be determined.

### Survival

During these experiments, all animals survived the inflicted pTBI, and only 2 out of 65 animals had to be euthanized according to ethical guidelines due to bad overall condition manifesting as excessive weight-loss and scruffy fur. Our results confirmed the importance of the choice of the anesthetic agent, and the usefulness of isoflurane in neurotrauma research. Similar findings have been reported by Statler et al. describing a neuroprotective effect of isoflurane after CCI (28). The results of our pilot study showed that the anesthesia with fentanyl/fluniasone and midazolam (Hypnorm and Dormicum) mixture we previously used in our rat pTBI study (19) led to 50% of lethality in all conditions. This further suggests a relative sensitivity of mice to this anesthesia, and caution about its utilization in neurotrauma research.

### Bodyweight

Loss of bodyweight has been reported in mice after CCI (40), fluid-percussion (29), and blast-induced TBI (27) models. In contrast, the PBBI-model did not cause weight-loss in rats (18). In the current experiments, we did observe an initial weight-loss in all pTBI groups, but the mice gradually regained weight, and their bodyweight was not statistically different from controls at the end of the experiment.

### Motor function

Motor coordination was assessed with the rotarod test, since it has been described as a more sensitive and efficient method than the beam-walking or – balancing tests in detecting motor impairment after brain injury in rodents (24). Spontaneous recovery is commonly seen after experimental TBI (18, 24), although the exact biological mechanisms underlying this phenomenon still need further clarification. In the accelerating rotarod test, rats subjected to PBBI (41) and mice injured by lateral CCI (42) showed an initial impairment, followed by gradual recovery to levels comparable to pre-injury after 7 days. This was also the case in the current study for mice subjected to 35- or 50-bar loading-pressure; however, the animals subjected to 100-bar loading-pressure still had a significant impairment at 7 days post-injury. Future studies with longer observation post-injury period are needed to verify whether this motor deficit is permanent. This would be particularly interesting for comparing two mouse TBI models; namely, we have previously observed that mice subjected to mild- or moderate-intensity blast-TBI manifested a significantly reduced rotarod-performance from day 1 to 7 days post-injury, but by day 14, they had recovered to pre-injury levels (27).

### Neuronal injury

We observed a progressive expansion of the injured area over the 7-day observation period, when a central cavity was observed. This progressive nature of injury will be examined in future studies, and could form a platform for investigating novel neuroprotective treatments. In the present study, positive Fluoro-Jade staining demonstrating on-going degeneration of neurons in the cerebral cortex was observed at 24 h post-injury, rapidly declining thereafter. This time-course is in line with previously published results using the PBBI-model (43) or our rat pTBI model (19). Early neurodegeneration could potentially be a signature of the penetrating type of TBI, since Fluoro-Jade staining seems to persist longer, i.e., beyond 24 h in the CCI model (44, 45). Given that many TBI cases contain elements of both focal and diffuse injury types, it has been argued that animal models that fail to generate pathological features of diffuse axonal injury (DAI) are of questionable value (21). In our model, we have observed one DAI hallmark: the accumulation of the βAPP in white matter tracts (Figure 3F). Although, we have previously reported βAPP-staining as early as 3 h post-injury in our rat version of the pTBI model (20), further and more detailed analyses are needed to define the features of DAI in this model.

### Hemorrhage and blood–brain-barrier defects

Hemorrhage and BBB-defects were noted in the lesion area identified by IgG immunohistochemistry and by the presence of erythrocytes. BBB-defects, edema, and hemmorhage can all increase the intracerebral pressure (ICP). Thus, our findings further emphasize the need for ICP monitoring in experimental models of TBI. Indeed, infrequently used ICP measurement in experimental TBI models has been identified as one of the important discrepancies between clinical and experimental TBIs, which could contribute to difficulties in transferring results from experiments to the clinical arena and *vice versa* (35, 46, 47). Hemorrhage can also cause oxidative injury induced by lysed erythrocytes releasing hemoglobin and iron (48–50). The importance of hemorrhage-induced reactive oxygen species in pTBI could be further elucidated in future, using several mouse-strains deficient in pathways involved in protection against oxidative stress (21).

### Astro- and microgliosis

Using our previously described method of semi-quantitative measurements of GFAP and Iba1 (19), we analyzed the temporal profile of glial activation in the cortex of mice with pTBI. The statistically significant increase in GFAP immunoreactivity, observed at 72-h and persisting up to 1-week post-injury, was comparable to our findings in the rat pTBI model (19). In contrast, the PBBI-model developed by Williams and co-workers (18, 51) generated a somewhat different time-course of astrogliosis. Namely, in their experiments, GFAP was up-regulated already at 6 h post-injury, peaking at 72 h, whereas only a minor staining level was observed at 7 days post-trauma (51). While the exact reason for this difference is currently unclear, we suggest methodological differences in injury induction (inflated balloon in the Williams model versus propelled projectile in ours) as the most plausible explanation. Reactive astrocytes perform both beneficial and detrimental functions following central nervous system injury (52). The protective functions include removal of excessive amounts of extracellular glutamate (53), resealing the BBB (54), and supporting axonal regeneration (55, 56). Astrocytes may also aggravate injury by forming scar tissue that contains inhibitory molecules of axonal regrowth (57, 58) and produces proinflammatory cytokines (59). After penetrating injury, astrocytes up-regulate the expression of aquaporin-4 (60), which contributes to edema formation (52). We also analyzed the microglia/macrophage reaction by using Iba1 immunohistochemistry. Cortical microglia remained activated up to 7 days post-injury, similar to our previous results in the rat pTBI model (19). However, this activation was longer that the reactive response described in the PBBI-model (51), where at 7 days, only minor levels of microgliosis remained. Similar to astrocytes, activated microglia can perform both protective and harmful tasks such as scavenging, phagocytosis, antigen presentation, synaptic stripping as well as secretion of both pro- and anti-inflammatory mediators (61, 62).

## Conclusion

In conclusion, we have developed a model for pTBI that reproduces non-fatal penetrating injury in mice. The three levels (i.e., 35-bar, 50-bar, or 100-bar loading-pressures) of the injurious factor (i.e., penetrating probe) were defined in terms of the probe’s speed, kinetic energy of the impact, and the size of the damage (i.e., cavitation). These measures were thereafter used to establish a relationship between the intensity of the injurious factor and the severity levels (i.e., mild, moderate, or severe) of the related *in vivo* injuries measured through several pathological reactions such as tissue destruction, neurodegeneration, gliosis, and motor performance. In most outcome measures, the most severe injury level induced by the 100-bar loading-pressure significantly differed from the mild or moderate injury severities induced by 35-bar or 50-bar loading-pressures, respectively. Nevertheless, no significant differences in the outcome parameters were found between the mild and moderate pTBI groups of mice. For further characterization and standardization, studies with longer observation periods, different injury locations, and/or additional and more sensitive functional tests are required. Finally, it remains to be determined whether the current mouse pTBI model reproduces cognitive impairments such as attention- and reference memory deficits comparable to those we have observed in our previous rat pTBI model (19).

## Conflict of Interest Statement

The authors declare that the research was conducted in the absence of any commercial or financial relationships that could be construed as a potential conflict of interest.

## Supplementary Material

The Supplementary Material for this article can be found online at http://www.frontiersin.org/Journal/10.3389/fneur.2014.00209/abstract

Video 1 **Gel impact at 35 bar loading pressure**.

Video 2 **Gel impact at 50 bar loading pressure**.

Video 3 **Gel impact at 100 bar loading pressure**.

## Footnotes

^1^http://www.gelatininnovations.com/pages/ballistic_fast_ins.html

^2^http://rsbweb.nih.gov/ij/
